# Stress, Resilience, and Well-Being in Italian Children and Their Parents during the COVID-19 Pandemic

**DOI:** 10.3390/ijerph17228297

**Published:** 2020-11-10

**Authors:** Maria Cusinato, Sara Iannattone, Andrea Spoto, Mikael Poli, Carlo Moretti, Michela Gatta, Marina Miscioscia

**Affiliations:** 1 Pediatric Diabetes Unit, Department of Women’s and Children’s Health, Padua University Hospital, 35128 Padua, Italy; maria.cusinato@phd.unipd.it (M.C.); carlo.moretti@aopd.veneto.it (C.M.); 2 Child and Adolescent Neuropsychiatry Unit, Department of Women’s and Children’s Health, Padua University Hospital, 35128 Padua, Italy; sara.iannattone@studenti.unipd.it (S.I.); michela.gatta@unipd.it (M.G.); 3 Department of General Psychology, University of Padua, 35131 Padua, Italy; andrea.spoto@unipd.it; 4 Department of Developmental Psychology and Socialization, University of Padova, 35131 Padua, Italy; Mikael.poli@studenti.unipd.it

**Keywords:** COVID-19, quarantine, children resilience, children well-being, parenting stress, parents well-being, COVID-19 pandemic

## Abstract

The novel coronavirus (COVID-19) outbreak has forced parents and children to adopt significant changes in their daily routine, which has been a big challenge for families, with important implications for family stress. In this study, we aimed to analyze the potential risk and protective factors for parents’ and children’s well-being during a potentially traumatic event such as the COVID-19 quarantine. Specifically, we investigated parents’ and children’s well-being, parental stress, and children’s resilience. The study involved 463 Italian parents of children aged 5–17. All participants completed an online survey consisting of the Psychological General Well Being Index (PGWB) to assess parental well-being, the Strengths and Difficulties Questionnaire (SDQ) to measure children’s well-being, the Parent Stress Scale (PSS) to investigate parental stress, and the Child and Youth Resilience Measure (CYRM-R) to measure children’s resilience. The results show that confinement measures and changes in daily routine negatively affect parents’ psychological dimensions, thus exposing children to a significant risk for their well-being. Our results also detect some risk factors for psychological maladjustments, such as parental stress, lower levels of resilience in children, changes in working conditions, and parental psychological, physical, or genetic problems. In this study, we attempted to identify the personal and contextual variables involved in the psychological adjustment to the COVID-19 quarantine to identify families at risk for maladjustment and pave the way for ad hoc intervention programs intended to support them. Our data show promising results for the early detection of the determinants of families’ psychological health. It is important to focus attention on the needs of families and children—including their mental health—to mitigate the health and economic implications of the COVID-19 pandemic.

## 1. Study Rationale

The novel coronavirus (COVID-19) rapidly spread in China, reaching several countries in a short amount of time [1]. Italy was the second country affected by the COVID-19 pandemic outbreak: more than 100,000 people (including at least 8956 health-care workers) have been infected and over 11,000 have died [2] (data updated on 2 April 2020). To control and prevent disease transmission, many countries have implemented mass quarantine measures. Due to the rise in COVID-19 cases, on 9 March 2020, the Italian government issued a decree imposing lockdown measures on the country, intending to prevent the further spread of coronavirus in both the most- (e.g., Lombardy) and least-affected areas. In addition to closing all non-essential activities and suspending all public gatherings, the decree banned people from leaving their house unless strictly necessary, under penalty of severe sanctions (e.g., fines and imprisonment), until 3 April. The containment measures were then extended and progressively lifted until 13 April and finally until 3 May. Mass quarantine (also known as “lockdown”) is the primary tool used by governments to manage an epidemic outbreak, forcing citizens living in affected areas to stay inside their house, only leaving in a few selected circumstances, e.g., for medical examinations, to look after a vulnerable person, buy essential goods like pharmaceuticals, food, and beverages, or carry out essential work. Any other type of non-essential activity is therefore suspended or carried out from home through “smart (remote) working” [3]. Such drastic changes, which include bans on a wide range of activities, school closures, travel restrictions, social distancing, and the rapid deterioration of local businesses, have generated a considerable amount of stress among the population. Stress during lockdown was also associated with amplified media coverage of the COVID-19 pandemic, and with both the transformation of work and education models and substantial general lifestyle alterations for millions of people all over the world [4].

## 2. Children’s Well-Being and Resilience during Quarantine

Studies conducted on the adult population have highlighted that social isolation measures might negatively affect psychological health [5]. People in quarantine are more likely to exhibit psychological symptoms and disorders, such as anxiety, anger, sleep disorders, depression, and post-traumatic stress disorder (PTSD) [6].

Because of the serious psychological implications of school closures and significant changes in daily routine, quarantine could have traumatic consequences not only on the adult population but on the pediatric one too [7].

Specifically, previous research has demonstrated that children who were subjected to quarantine and social isolation show higher levels of post-traumatic stress disorder (PTSD) compared to children who did not experience such measures [8]. Research conducted during the COVID-19 pandemic highlighted that children and adolescents were more likely to manifest externalized symptoms, such as inattention, persistent inquiry, irritability, and internalized symptoms, such as anxiety and depression or hyperactivity [9,10]. Moreover, children and adolescents exposed to traumatic events presented anxiety, depression, lethargy, reduced appetite, and emotional and behavioral difficulties several years after the events [11,12].

Nevertheless, several personal and interpersonal variables such as age, personality, developmental level, cognitive and emotional abilities, coping strategies, and resilience may influence children’s reactions to disaster [13,14], as well as parents’ responses and family support systems [15].

Recent studies have shown that, during potentially traumatic events, some people can adaptively face negative circumstances, with mild or moderate reactions that do not interfere with their functional abilities [16,17,18]. The ability to thrive despite exposure to adversity is known as resilience. In a social–ecological framework, resilience depends on the quality of the interactions with the environment, which allows individuals to maintain their psychological, social, and physical well-being [19,20,21,22].

Children’s adjustment to unknown situations is also influenced by distal factors, such as social changes and rules imposed during the COVID-19 pandemic, and proximal factors, including peer and infant–caregiver relationships [23]. The research on distal factors suggests that children and their parents constitute an “at-risk group” for maladjustment due to lifestyle disruption caused by COVID-19 [24]. In fact, children may be more sensitive to routine changes compared to the general population [25]. The significant changes in daily routine that children have experienced during quarantine might interfere with their sense of predictability and security—both essential factors for healthy development [26]. Indeed, these modifications expose children to a higher risk for negative health outcomes resulting from lower levels of physical activity, longer screen time, irregular sleep patterns, and poorer eating habits [27]. In particular, school routine constitutes an important coping mechanism for children, allowing them to schedule their time, interact with their peers, and focus on achieving bigger and bigger goals [28].

Concerning proximal factors, it is important to highlight that children’s well-being is strictly linked to their parents’ physical, psychological, and social health [29]. A recent study showed how parental presence may decrease the child’s stress levels during quarantine [25].

## 3. Parents’ Well-Being and Parental Stress

Like their children, adults have faced different stressors linked to the quarantine period as well. Specifically, parents have had to cope with social distancing and changes in their daily routine, such as remote working or unemployment, as well as caring for their children during school hours. In some cases, the changes brought about by lockdown measures were accompanied by a reduced income and new responsibilities that may exacerbate pre-existing difficulties and stress [30,31,32].

Because of the COVID-19 outbreak and the resulting need for social distancing, many organizations have imposed a remote work policy, forcing individual citizens to work from home [33], therefore gradually blurring the boundaries between work and family roles [34]. At the same time, the closing of schools and childcare facilities has forced many working parents to take on full-time responsibility for their children’s care and home education, while simultaneously adapting to a new “smart working” lifestyle and daily structure. Parents found themselves having to manage their time during quarantine while balancing personal life, work, and their children’s education without any external help. This situation has put parents at increased risk for personal distress, potentially compromising their own well-being and, as a result, their children’s well-being [32].

The effects of the COVID-19 pandemic on families vary considerably based on what contextual stress factors parents are exposed to; data indicates that the consequences of lockdown tend to negatively affect low-income families the most [35]. More specifically, parental stress represents a negative psychological response to parental obligations: caring for children while simultaneously worrying about not having enough resources to meet their needs can be a heavy burden for parents [36,37,38]. Several personal characteristics of both parents and children in a specific family configuration, together with various life stressors, may contribute to an increased sense of psychological pressure resulting from the caregiving role [39]. Previous research has identified different parental factors that may increase the risk of parental stress: low confidence in one’s own parental abilities, low perceived attachment to the child, health problems, role restriction (i.e., the extent to which the parental role restricts the parents’ freedom and ability to maintain their own individual identity), depression, anxiety, and low levels of emotional and practical partner involvement (particularly, single parenting) [40,41].

As is well-known, parental stress is associated with children’s behavioral and emotional problems and developmental maladjustment [42]. During infancy, parental stress can negatively alter parent–child interactions and interfere with the quality of caregiving, and, therefore, affect the formation of attachment bonds [43]. Moreover, parental stress is associated with higher levels of family conflict until adolescence [44,45], and, through parental behavior, it contributes to the creation of a chaotic family environment, which in turn, puts the child at risk for behavioral problems [46]. In addition, high levels of parental stress are thought to interfere with the caregiver’s ability to effectively cope with parenting-related difficulties [47], which may result in increased use of inappropriate disciplinary strategies and a higher risk of physical abuse [48,49].

## 4. Study Aims

The purpose of the present study was to investigate parents’ and children’s well-being, parental stress, and children’s resilience during the COVID-19 pandemic, more specifically during the quarantine.

Furthermore, we also aimed to investigate which proximal and distal variables constitute protective and risk factors for psychological maladjustment.

In particular:

(a) the first aim of our study was to evaluate children’s well-being during the quarantine. According to the literature, we expected to observe lower levels of psychological well-being in quarantined children compared to the normal population [8,25]. Moreover, we expected to observe lower levels of psychological maladjustment in children who presented high levels of resilience [22].
(b) the second aim of our study was to assess parents’ psychological adjustment during quarantine and the related parental stress. Controlling for socio-demographic variables [41], we expected to detect lower levels of psychological well-being in quarantined parents compared to the normal population [6]; we also expected higher parental distress to be associated with lower levels of well-being in both parents and children [32,42]. We expected to observe lower levels of psychological maladjustment and parental stress in parents whose children showed higher levels of resilience [19,20].
(c) finally, our third aim was to investigate the role that changes in parents’ working conditions and parents’ perception of their relationship with their children during quarantine can play on parental stress and both children’s and parents’ well-being.

## 5. Method

### 5.1. Participants and Procedure

To conduct the survey, we developed an ad hoc online questionnaire, which was divided into six areas: general information (i.e., region of residence, house dimension, family components), parent’s well-being, parental stress, children’s psychopathological symptoms, children’s resilience, and final questions (e.g., parents’ perception of the relationship with their child, parents’ degree of satisfaction with the relationship). Data collection began on 25 April 2020 and ended on 8 May 2020. The study was approved by the University of Padova Ethics Committee for Psychological Research (2020/num 3590), and all respondents expressed their willingness to participate via an informed consent form. A single link was sent only once to each potential respondent’s family. We asked participants that only one family member complete the survey, and we did not receive multiple responses from the same family. As such, the fathers and mothers who completed the survey are not associated.

We considered data collected from parents who had at least one child aged 5 to 17 years old living at home and who answered both child behavior questionnaires in their entirety—that is, the Strength and Difficulties Questionnaire (SDQ) and the Child and Youth Resilience Measure (CYRM-R). The sample size was established using the G*Power 3.1 software [50] to have a power of 0.95 in reliably detecting an effect of 0.09, with a type I error of 0.05.

Based on these inclusion criteria, the final sample consisted of 463 Italian-speaking parents (90.5% women) aged 29 to 64 (mean age = 43.3, SD = 5.88). In terms of location, 82.4% of the participants were from northern, 11.7% from central, and 5.9% from southern Italy. The number of children living at home ranged from one to four; the mean age of the oldest was 12.1 (SD = 5.44), while the mean age of the youngest was 8.22 (SD = 3.96). Participants were asked to think about one of their children—who were male in 56.2% of cases, with a mean age of 9.72 (SD = 3.29)—and answer accordingly. This choice was driven by our goal of conducting a sustainable research study, with the participants having enough time to complete the survey as the main factor in our decision. We also considered the large number of survey studies conducted in the same period by different University Departments.

Among the parents, 17.3% had one or more children under 5 years of age, 9.3% reported having at least one child suffering from a psychological, medical, or genetic disease, and 19.2% declared they suffered or had suffered from a condition related to one of these diseases. Regarding the family structure, 87.7% of families were nuclear (i.e., two parents and children), 6.7% included a single parent (i.e., the children were being raised by one parent only), 4.1% were blended (i.e., a parent raises his/her children with a partner who is not their biological parent; the partner may, in turn, have children from a previous relationship) and 1.5% were extended (i.e., many relatives living together). The urban areas where participants lived can be found in Table 1, whereas their educational level is reported in Table 2.

At the time of the study, the average duration of confinement was 50.6 days (SD = 20.7). As for daily routine modifications, 63.9% of the parents had changed their working conditions; more specifically, 45.2% worked from home (teleworking), 27.6% were unemployed or fired, 21.2% had a temporary interruption to their job, and 6.0% worked part-time. As for children, 94.6% continued their school activities from home. The hours that parents spent with their children increased from an average of 6.75 (SD = 3.79) before quarantine, to an average of 14.5 (SD = 7.11); in fact, 80.8% of the respondents took care of their children without any help from other people, such as relatives or babysitters. Lastly, 5.2% of parents had to move away from their children during lockdown due to health reasons, 6.7% reported at least one positive COVID-19 case in their family, and 7.2% had lost one or more relatives to the virus.

### 5.2. Measures

The Parent Stress Scale (PSS; [51]) is composed of 18 items focusing on three main parenting themes: “positive emotional benefits”, “sense of enrichment and personal fulfilment”, and “negative components” (ibidem). Parents were asked to answer according to their relationship with their children, using a five-point scale, where 1 = “strongly disagree” and 5 = “strongly agree”. The questionnaire provides a total score that represents the global level of parental stress. It has good reliability and validity, with a Cronbach’s alpha coefficient of 0.83 and test–retest reliability correlation of 0.81 over 6 weeks. This measure has been translated by our team.

The original Psychological General Well Being Index (PGWB; [52]) consists of 22 items, rated on a six-point scale, to which subjects were asked to answer referring to the last four weeks. The items reflect the following subscales: anxiety, depression, positive well-being, self-control, general health, and vitality. The sum of each score provides a global summary score, which represents the subjective perception of well-being or disease; in each scale, low scores correspond to lower levels of well-being. All American studies conducted on the PGWB found this instrument to have good internal consistency, with a Cronbach’s alpha coefficient ranging from 0.90 to 0.94.

The Italian version of the same questionnaire [53] has acceptable reliability and internal consistency, with a Cronbach’s alpha coefficient for each scale ranging from 0.61 to 0.85, and item-scales correlations ranging from 0.43 to 0.67. In our survey, we adopted the Italian short form of the PGWB (PGWB-S; [54]), composed of six items (n° 5, 6, 7, 18, 20, 21 of the original scale) that generally explain more than 92% of the global variance of the questionnaire. The PGWB-S showed good internal consistency, with a Cronbach’s alpha coefficient ranging from 0.80 to 0.92. In addition, we also considered items 3, 4, 8, 11, 14, 17, 19, 22 to complete the anxiety, depression, and self-control scales. The scale-level correlation matrix with the PGWB anxiety, depression, self-control scales, and the total score of PGWB-S, considering our sample, is shown in Appendix A Table A1.

To assess children’s well-being, we administered the Strengths and Difficulties Questionnaire (SDQ; [55])—Italian version [56]. The questionnaire consists of 25 items divided into five subscales: emotional symptoms, hyperactivity-inattention, conduct problems, peer relationship problems, and prosocial behavior. Parents were asked to answer based on their child’s behavior, using a three-point scale (“not true”, “somewhat true” and “certainly true”). The scores in each subscale (except for the prosocial behavior scale) can be summarized into a total score that reflects emotional and behavioral difficulties. The Italian version of the SDQ has good psychometric properties, with a Cronbach’s alpha ranging from 0.73 to 0.89.

Lastly, to assess children’s resilience, we used the Child and Youth Resilience Measure (CYRM-R; Resilience Research Centre, 2018)—Person Most Knowledgeable (PMK) version, translated into Italian by our team (using back-translation). The instrument consists of 17 items divided into two subscales: personal resilience (composed of 10 items) and caregiver/relational resilience (composed of 7 items).

In addition to these subscales, an overall “individual resilience” score is included. In the version of the questionnaire we adopted in this study, parents were asked to answer in relation to their child using a five-point scale, where 1 = “not at all” and 5 = “a lot”.

### 5.3. Data Analysis

Data were analyzed using the statistical software jamovi 1.1.9 [57].

First, we calculated descriptive statistics and frequency tables to highlight the characteristics of the population under study.

Second, since the Italian validations of the PSS and the CYRM-R are not yet available, we conducted reliability analysis and confirmatory factor analysis (CFA) to establish the applicability of these two questionnaires in their original factorial structures in the Italian context. As a result, two CFA models were fitted to the data. The goodness of fit for each of the two models was tested using the following fit indexes: the ratio between chi-square and degrees of freedom (*χ*^2^/df); the Comparative Fit Index (CFI; [58]); the Tucker Lewis Index (TLI; [59]); the Standardized Root Mean Square Residual (SRMR; [60]); the Root Mean Square Error of Approximation (RMSEA; [61]). The following criteria were used to indicate acceptable model fit: *χ*^2^/df < 3 (e.g., [62]), values close to 0.95 for CFI and TLI, values lower than or equal to 0.08 for SRMR and 0.06 for RMSEA [63]. In the CFA of the CYRM-R, we also considered the Bayesian Information Criterion (BIC; [64]) and Akaike’s Information Criterion (AIC; [65]) to compare the original factorial structure with the single-factor one. To estimate the internal consistency of the scales, we computed McDonald’s omega coefficient (*ω*; [66]) and item–rest correlations. Since we considered a wide age range for children (5–17 years), we wanted to observe whether the levels of children’s well-being and parental stress differed across age groups, as suggested by the literature. For this reason, we divided the sample into two subgroups: the first included children aged 5 to 12; the second included children aged 13 to 17. Then, we conducted two independent sample *t*-tests entering the total scores of the SDQ and PSS as dependent variables and the children’s age subgroups as independent variables. To test for the presence of statistically significant differences between the Italian normal population and our sample in terms of parents’ and children’s well-being, we computed one-sample *t*-tests, entering the means of the normative samples of the SDQ and the PGWB as test values. Given that only parents of children aged 6 to 10 were considered in the analysis for the Italian validation of the SDQ, in this *t*-test, we included only those participants whose children’s ages were within that range (*n* = 40). As for the PGWB, we conducted two different *t*-tests for mothers (*n* = 419) and fathers (*n* = 44).

To verify whether socio-demographic variables affected parental stress and well-being, we fitted two general linear models (GLM). Specifically, in the first GLM, the variables were organized as follows: the total score of the PSS was the dependent variable, while the presence in the family of children younger than five (two levels), the family structure (four levels), the presence of modifications in working conditions (two levels), and the presence of parents and children with genetic, physical, or psychological disorders (two levels) were the independent variables; furthermore, the number of children living with parents and the child’s age were covariates. In the second GLM, the total PGWB score was the dependent variable, whereas the presence of children younger than five, modifications in working conditions, and parents and children with genetic/physical/psychological conditions were the independent variables; the child’s age was the covariate.

To test whether children’s resilience, children’s well-being, and parents’ psychological condition influenced the level of parental stress, we conducted a multiple linear regression, with the total PSS score as the dependent variable and the total scores of the PGWB, SDQ, and CYRM-R as predictors.

We performed an analysis of variance (ANOVA) to verify whether parents’ different perception of how the quarantine period changed their relationship with their children (three levels) affected their level of stress, considering the total score of the PSS as the dependent variable. Post hoc tests were conducted using the Bonferroni correction.

Subsequently, to establish the effects of changes in work routine due to COVID-19 on children’s well-being, we conducted independent sample *t*-tests, considering the SDQ scales as dependent variables and the presence or absence of modifications in working conditions (two levels) as the independent variable.

Along the same lines, independent sample *t*-tests were computed to test statistically significant differences between (i) the well-being of male and female children; (ii) the mothers’ and fathers’ observations of their children’s behavioral and psychological symptoms.

Lastly, we ran correlation tests using Pearson’s r coefficient to explore the relationships between children’s resilience, their behavior, and parents’ stress and psychological condition.

Considering the explorative nature of the present study, the threshold for significance was set at *p* < 0.01, to reduce the probability of reporting and considering spurious findings.

## 6. Results

### 6.1. Factorial Validity and Reliability of the Italian Version of the PSS and CYRM-R

The first reliability analysis of the PSS revealed that the item–rest correlation of item 2 was the lowest (r = 0.08). We checked the item’s content and noticed a problem with phrasing, so we decided to remove the item before conducting the remaining analyses. The CFA showed an acceptable goodness of fit of the structure of the questionnaire: *χ*^2^ (104) = 263, *p* < 0.001, *χ*^2^/df = 2.53; CFI = 0.930; TLI = 0.908; SRMR = 0.052; RMSEA = 0.059. Absolute standardized factor loadings were all significant, ranging from 0.17 to 0.72. Regarding the internal consistency indexes, item–rest correlations varied from r = 0.18 to r = 0.66, while McDonald’s *ω* was 0.87 for the full scale and between 0.85 and 0.87 by removing one item at a time.

As for the CYRM-R, Table 3 shows the fit indexes of the first CFA, conducted maintaining the two different factors found in the original version. Since, in our survey, we used the PMK version of the questionnaire—that is, completed by parents about their children—we carried out another CFA testing a single-factor structure of the instrument. This further analysis revealed better fit indexes compared to the previous one (Table 3) and significant standardized factor loadings, ranging between 0.30 and 0.71, so we decided to consider the single-factor structure. Moreover, reliability analyses demonstrated good internal consistency indexes: item-rest correlations ranged from r = 0.27 to r = 0.65; McDonald’s *ω* was 0.86 for the full scale, while it varied between 0.84 and 0.86 by removing one item at a time.

### 6.2. Children’s Well-Being and Resilience

Concerning the differences between children’s age subgroups in the SDQ’s total score, the result of the independent sample *t*-test was not statistically significant (t_461_ = 0.937, *p* = 0.354; M_5–12_ = 11.35, SE = 0.319; M_13–17_ = 10.73, SE = 0.587), indicating that we could include all children into a single age group.

The one-sample *t*-test, computed using the mean scores of the Italian normative sample of the SDQ as test values, did not show any statistically significant difference between the normative sample’s scores and our children’s (aged 6–10) scores.

Regarding the differences between male and female children in the SDQ’s scales, the results of the independent sample *t*-test indicated that the children’s gender only significantly affected the prosocial behavior scale (t_455_ = −4.12, *p* < 0.001), with females obtaining higher scores than males (M*_boys_* = 7.26, SE = 0.117; M*_girls_* = 7.94, SE = 0.116).

Considering the relationship between children’s resilience and their well-being, the results show significant Pearson’s r correlations between the CYRM-R total score and the SDQ scales: we observed negative correlations with all SDQ scales (values ranging from r = −0.40 to r = −0.63), except for the prosocial behavior scale (r = 0.43).

Lastly, concerning parents’ opinions about their children’s well-being, the *t*-test highlighted significant differences between mothers and fathers only in the SDQ emotional symptoms scale (t_64.3_ = 4.06, *p* < 0.001); specifically, mothers obtained a higher average score (M = 2.85, SE = 0.102) than fathers (M = 1.89, SE = 0.214).

### 6.3. Parental Well-Being and Stress

In analyzing the level of parental stress as a function of the children’s age subgroups, the independent sample *t*-test did not show any statistically significant difference in the PSS total score (t_445_ = 0.457, *p* = 0.676; M_5–12_ = 35.6, SE = 0.468; M_13–17_ = 35.2, SE = 0.925). This result allows us to include all parents in a single group.

The one-sample *t*-test, computed considering the means of the Italian normative sample of the PGWB as test values, showed that the mothers in our sample obtained significantly lower average scores compared to those of the normative sample in the following scales: total score (t_412_ = −14.3, *p* < 0.001; M*_normative_* = 19.76; M*_sample_* = 17.0, SE = 0.192), anxiety (t_412_ = −6.40, *p* < 0.001; M*_normative_* = 16.6; M*_sample_* = 15.1, SE = 0.231), and self-control (t_412_ = −5.63, *p* < 0.001; M*_normative_* = 11.5; M*_sample_* = 10.7, SE = 0.136). Conversely, no significant differences emerged when the male sample was analyzed.

As for the socio-demographic factors influencing parental stress, the first GLM, with the total PSS score as the dependent variable, was significant overall (F (9, 428) = 4.818, *p* < 0.001; R^2^ = 0.092, adjusted R^2^ = 0.073). In this model, significant effects were observed for family structure (F (3, 428) = 4.73, *p* = 0.003, ω^2^ = 0.024), number of children living at home (F (1, 428) = 6.50, *p* = 0.011, ω^2^ = 0.012), and presence of children with psychological, physical or genetic disorders (F (1, 428) = 15.58, *p* < 0.001, ω^2^ = 0.031).

With respect to family structure, the post hoc test with Bonferroni correction showed a significant difference between the nuclear family structure and the single-parent structure, with the members of the latter obtaining higher average scores (t_428_ = −3.68, *p* = 0.002; M*_single−parent_* = 43.1, SE = 1.818; M*_nuclear_* = 36.9, SE = 0.871). Moreover, participants who changed their working routine showed higher levels of stress (M = 40.4, SE = 1.32) compared to those whose working conditions remained unchanged (M = 38.5, SE = 1.40). Participants living with children affected by physical or psychological problems also obtained higher PSS scores (M = 42.3, SE = 1.74) compared to the parents of healthy children (M = 36.6, SE = 1.14). In addition, parental stress levels increased as a function of the number of children living at home (*β* = 0.12, *p* = 0.011).

Concerning the socio-demographic factors affecting parental well-being, the second GLM with the total PGWB score as the dependent variable was significant overall (F (5, 451) = 8.761, *p* < 0.001; R^2^ = 0.089, adjusted R^2^ = 0.078). This variable seemed to be significantly affected by the presence of modifications in parents’ working conditions (F (1, 451) = 18.97, *p* < 0.001, ω^2^ = 0.036), the child’s age (F (1, 451) = 6.61, *p* = 0.010, ω^2^ = 0.011), and the presence of parents suffering from psychological, physical or genetic problems (F (1, 451) = 13.61, *p* < 0.001, ω^2^ = 0.025).

In particular, parents who suffered/had suffered from medical or psychological conditions obtained lower scores (M = 15.9, SE = 0.487) compared to those who did/had not (M = 17.5, SE = 0.356), reflecting lower levels of well-being. As for working conditions, participants who changed their working routine showed lower PGWB total scores (M = 15.9, SE = 0.383) compared to those whose working condition was unchanged (M = 17.5, SE = 0.436). Lastly, both PGWB scores and parental well-being increased as a function of the child’s age (*β* = 0.12, *p* = 0.010).

The multiple linear regression showed that the total scores of the PGWB, SDQ, and CYRM-R were all significant predictors of parental stress levels. In particular, the total PGWB and CYRM-R scores were negative predictors of parental stress (respectively: *β* = −0.28, t = −6.37, *p* < 0.001 and *β* = −0.21, t = −4.17, *p* < 0.001), while the total SDQ score was a positive predictor (*β* = 0.22, t = 3.99, *p* < 0.001). The overall R^2^ of the model was 0.32, while the adjusted R^2^ was 0.31.

As for the relationship between children’s resilience and parents’ well-being and stress, we performed a correlation test between CYRM-R total score, PGWB’s scales, and PSS total score. Correlations between the CYRM-R and the PGWB were significant, albeit low, varying from r = 0.22 to r = 0.27; conversely, the correlation between the CYRM-R and the PSS was significant and negative (r = −0.43).

### 6.4. Changes in Parents’ Working Conditions and Parents’ Perception of Their Relationship with Children

The ANOVA indicated that different perceptions of how the quarantine period influenced the parent–child relationship significantly affected parental stress (F (2, 444) = 14.3, *p* < 0.001). The post hoc test with Bonferroni correction showed significant differences between the none–positive and negative–positive levels of the independent variable (Table 4): parents who experienced the changes in their relationship with their children in a negative way obtained the highest average scores in the PSS (M = 39.4, SE = 0.966), whereas those who experienced such changes positively obtained the lowest average scores (M = 33.6, SE = 0.585).

Concerning the influence of modifications in parent’s working conditions on children’s well-being, the independent sample *t*-test showed that the presence of changes in the working routine did not seem to significantly affect any SDQ scale.

## 7. Discussion

In our study, we aimed to investigate the psychological impact of COVID-19 quarantine rules on Italian children aged 5–17 and their parents, and to unveil the contextual and personal variables associated with better overall adjustment.

To the best of our knowledge, this study is one of the few [9,32,67,68] to investigate the well-being of the parent–child dyad including the role of children’s resilience and parental stress as protective and risk factors, respectively. Due to the COVID-19 pandemic, many families faced several changes in their everyday life. Keeping their children engaged and safe at home represented a significant challenge (and sometimes a difficult task) for many parents, especially considering the psychological impact of quarantine on children.

Consistent with recent research findings, our data show that confinement measures and changes in daily routine negatively affect both children’s and parents’ behavioral and emotional dimensions [69].

Concerning our first specific aim—to evaluate children’s well-being and resilience during quarantine—our results do not indicate significant differences in terms of well-being between children (aged 6–10) compared to the normative population; however, regarding the hyperactivity scale of SDQ, the results of the one-sample *t*-test do not support the complete absence of such difference (t = 2.18, *p* = 0.036), suggesting that there might be higher levels of hyperactivity among the children during lockdown compared to the normal population (M*_normative_* = 3.30; M*_sample_* = 4.13, SE = 0.379). This result seems to be in line with recent studies that consider hyperactivity as a common psychological reaction during periods of confinement [9,10]. An important aspect linked to hyperactivity is the tendency for children to express their negative emotions through aggressive behaviors [70], sometimes developing externalized symptoms rather than post-traumatic stress disorder [17]. For these reasons, future research should take into consideration children’s hyperactivity to clarify and better investigate this reaction.

As for children’s well-being, we observed a negative association between children’s psychopathological symptoms and resilience. This result is consistent with the literature [22,71] and further highlights the notion that children who can thrive despite adversities and adapt to the imposed restrictions and social distancing can mitigate the negative impact of these aspects on their own well-being.

Concerning our second specific aim—to assess parents’ well-being and parental stress during the COVID-19 outbreak—our study shows that the mothers included in our sample (the majority of the respondents) report lower levels of well-being and perceived self-control and higher levels of anxiety compared to the normal population. Similarly to what has been observed in other countries [25,72], these findings confirm the negative psychological impact of the pandemic, and further highlight the presence of moderate to severe anxiety symptoms in Italian mothers during the lockdown. The quarantine measures imposed during the COVID-19 pandemic represent an uncertain and threatening situation that could trigger anxiety symptoms [73] and interfere with personal self-control [74].

In addition, results show that certain socio-demographic and contextual variables affect parental well-being and stress—changes in working conditions, younger children, and parental psychological, physical, or genetic problems appear to be linked to lower levels of psychological well-being in parents during the COVID-19 lockdown. Family structure, the number, and the presence of children with psychological, physical, or genetic diseases are all risk factors for high parental stress.

Our findings are consistent with the literature highlighting how disabilities or chronic conditions represent a high-impact stress for parents [75,76,77,78,79]. Moreover, as expected, being part of a nuclear family constitutes a protective factor for stress and psychological sequelae in parents. Parenthood is a demanding role, particularly during periods of confinement such as quarantine, in which parents must manage multiple tasks, increasing the risk of role conflict [80]. In single-parent families, parents must balance different roles on their own; this aspect could negatively affect their psychological health, exposing them to maladjustment, especially during stressful situations [81,82].

According to our results and the literature, other family structure characteristics that could increase the level of parental stress include the number of children living at home. All these aspects point out that pre-existing vulnerabilities constitute risk factors for psychological sequelae in both parents and children during the COVID-19 outbreak, as further highlighted by our results. Moreover, an additional aspect worth considering is the impact that parental stress can have on parenting skills. High levels of parental stress are thought to interfere with the caregiver’s ability to effectively cope with parenting-related difficulties [47], which may result in an increased use of inappropriate disciplinary strategies and might lead to a higher risk of physical abuse [83] and neglect [84].

Nevertheless, some family resources can help protect both children and parents from the negative impact of stressful situations and promote their well-being, as a recent study has shown [85]. According to our results, one protective factor is children’s resilience, which, as discussed above, is strictly connected to their well-being. In addition, regarding the relationship between children’s resilience and parents’ psychological health, our hypothesis was only partially confirmed: the correlations between the CYRM-R and the PGWB scales, although significant, are low. Furthermore, our results show a negative association between children’s resilience and parental stress: this indicates that the first may mitigate the latter. All these aspects emphasize the role of resilience in the family context, as well as the mutual influence that children and parent’s psychological health exert, and the ability to successfully face life’s challenges.

Children’s well-being represents an additional negative predictor of parental stress. For this reason, it may constitute a protective factor for parental distress, meaning that parents of children with better psychological adjustment experience fewer difficulties in their parental role. In this sense, all family members reciprocally influence each other’s adjustment, and can help develop new resources by promoting positive adaptation during difficult times.

Finally, regarding our third specific aim—to analyze the effects of lifestyle changes linked to the COVID-19 pandemic on parents’ and children’s psychological health—changes in working conditions seem to play a crucial role in the family’s psychological adjustment. Data show that parents who change working conditions suffer from psychological difficulties more than those who do not. Such changes include job loss or temporary suspension of work activities, which imply a reduction in income, with negative consequences for psychological and physical adjustment [86,87]. Similarly, performing one’s job through remote working constitutes another risk factor for maladjustment.

It was quite difficult for parents to balance their parental role and their work-life, with important implications for psychological health [88]. The impact of such changes must not be underestimated. Regarding the family context: maintaining clear family and work boundaries can be more difficult during the lockdown, as people must now undertake multiple role transitions during their day [89]. Although permeable family and work boundaries can represent an advantage [90], taking on different competing roles (e.g., parent and employee) can lead to conflict between them, family and work interferences, and emotional tension [91]. During quarantine, parents had to manage their work while at the same time helping their children with their homework, often without the possibility to occupy separate rooms.

In our survey, parents were asked to think about their relationship with their children during the lockdown. Some of them (18.1%) reported negative effects of the confinement measures on their interactions with their children, whereas the majority (48.8%) reported positive changes in the parent–child relationship (e.g., spending more time together). Our results show that the perception of how quarantine can transform the relationship with one’s children plays a key role in the stress experienced by parents. Those who perceived quarantine as an opportunity to spend more time with their children reported lower levels of parental stress. This aspect highlights the crucial role of the cognitive representation of an event in reducing the intensity of the psychological response to it. In particular, positive parental representations of the quarantine period help caregivers implement positive parenting styles, which in turn support children’s development and decrease the incidence of externalizing behaviors [69].

Despite the interesting findings that emerged from the present study, its limitations need to be addressed.

First, online surveys certainly have their advantages, such as granting the possibility to quickly reach a high number of participants in different areas of the country; however, using one did not allow us to control the contextual features of the environment where the assessment occurred (e.g., noises and interruptions may have distracted the participants). Second, the online procedure did not allow us to check whether participants accurately completed the questionnaire in its entirety, following the instructions from beginning to end. Third, there is a possible sampling error linked to the request to take into consideration only one child for each family. On the one hand, this allowed us to avoid any bias linked to the maximization or minimization of differences between siblings, due to the repetitive compilation of the same questionnaires; on the other hand, it was not possible to control parents’ answer choice criteria. These possible errors caused by online survey administration require caution in the interpretation of these results. Moreover, basic Internet and technical skills were required to participate in the study—such skills include being able to operate smartphones, computers, or tablets. Because of this, our study only included Internet users. Although this aspect may constitute a limitation, parents of children aged 5 to 17 frequently use technological devices to surf the Web. Statistics show that in Italy, in November 2019, 83% of people aged 35 to 44, and 81% of people aged 45 to 54 were daily Internet users. Considering that the mean age of the parents included in our sample was 43.3 years, it is safe to assume that accessing the online survey did not constitute a difficult task for them.

Lastly, our sample is not representative of fathers because only 9.5% of the responders belonged to this category.

Our study provides a general view of Italian families’ experience during the quarantine. Future longitudinal studies will allow a more exhaustive understanding of the real long-term effects of the COVID-19 outbreak on children’s and parents’ psychological adjustment and the entire family system.

Currently, in Italy, the daily situation update regarding the epidemiological curve shows a rise in contagion, which has led to the government tightening social distancing rules and general security measures. As such, those most at-risk will be the most vulnerable. In this current scenario, it will be important to provide psychological support to those who have already suffered from psychological maladjustments or have been experiencing difficulty in their role as a parent or in their family relationship during quarantine; implementing preventive activities and well-being programs to support a sense of competence, social connections, and parental resilience for the new challenges expected in this new phase will be crucial for public health.

The purpose of our study was to not only on risk factors, but also on individual and family strengths. Our study highlights the importance of supporting the most vulnerable groups, and points out that even during critical times, people can rely on their existing psychological resources, which must be further developed to maximize their potential. For these reasons, our study may represent a starting point upon which to build specific psychological treatments to help the population overcome this difficult period and limit long-lasting consequences on people’s psychological health.

## 8. Conclusions

Our findings point out that the COVID-19 quarantine constitutes a challenging period for Italian children and parents. In fact, changes in daily routine negatively affect parents’ psychological dimensions and expose children to a significant risk for their well-being. This study identifies some personal and contextual variables involved in the psychological adjustment to the pandemic that can help the healthcare system and health care professionals in the early detection of families at risk for maladjustment. The Government should focus attention on the needs of families and children and plan ad hoc programs for families to mitigate the health and economic implications of the COVID-19 pandemic.

## Figures and Tables

**Table 1:** Frequencies of the areas where the participants lived.

Urban Area ^1^	Participants *n* (%)
Village	57 (12.3%)
Countryside	108 (23.3%)
Small city	153 (33.0%)
Mid-sized city	115 (24.8%)
Large city	30 (6.5%)

^1^ To define the dimensions of the urban areas, we considered the density of the population, where the larger city has a population of at least 50,000 inhabitants, according to Italian territory.

**Table 2:** Frequencies of the educational level of the participants.

Educational Level	Participants *n* (%)
Primary	4 (0.9%)
Secondary	34 (7.3%)
High school	201 (43.4%)
University	167 (36.1%)
Post-university ^1^	57 (12.3%)

^1^ It is the highest educational level completed and it includes postgraduate specialization and PhD.

**Table 3:** Fit indexes of the original factorial structure of the Child and Youth Resilience Measure (CYRM-R) compared to those of the single-factor structure.

Model	*χ* ^2^	df	*p*	*χ*^2^/df	CFI	TLI	SRMR	RMSEA	AIC	BIC
Two factors	300	109	<0.001	2.75	0.914	0.893	0.050	0.062	20,364	20,616
One factor	276	107	<0.001	2.58	0.924	0.903	0.049	0.059	20,344	20,605

Legend: CFI: Comparative Fit Index; TLI: Tucker Lewis Index; SRMR: Standardized Root Mean; Square Residual; RMSEA: Root Mean Square Error of Approximation; AIC: Akaike’s Information.

**Table 4:** Results of the post hoc test considering how, according to parents, the quarantine period changed their relationship with their children.

Perceived	Change	Mean Difference	*t*	df	*p_Bonferroni_*
None	Negative	−3.20	−2.69	444	0.022
None	Positive	2.66	2.93	444	0.011
Negative	Positive	5.86	5.19	444	<0.001

## Appendix A

**Table A1:** *Scale*-level correlation matrix with the Psychological General Well Being Index (PGWB) anxiety, depression, self-control scales and the total score of the short form of the Psychological General Well Being Index PGWB-S considering our sample.

	TOT PGWB-S	Anxiety	Depression
**Anxiety**	0.86		
**Depression**	0.76	0.68	
**Self-control**	0.78	0.68	0.67
